# Large Mucocele of the Appendix Discovered in a 48-Year-Old Male Presenting With Appendicitis: A Case Report

**DOI:** 10.7759/cureus.33011

**Published:** 2022-12-27

**Authors:** Dimitar I Semerdzhiev, Robert E Hardister, Stephen D Wagner, Dewey R McAfee, David E Martin, Suporn Sukpraprut-Braaten

**Affiliations:** 1 Internal Medicine, Arkansas College of Osteopathic Medicine, Fort Smith, USA; 2 General Surgery, Unity Health, Searcy, USA; 3 Internal Medicine, Unity Health, Searcy, USA; 4 Graduate Medical Education, Unity Health, Searcy, USA; 5 Graduate Medical Education, Kansas City University, Kansas City, USA

**Keywords:** right colectomy, mucocele excision, appendectomy variants, atypical appendicitis, mucocele of the appendix

## Abstract

The appendix is a vermiform-like organ that extends from the cecum and has been thought of as having a rudimentary immunologic function. However, the appendix can become distended and mucus-filled, classifying it as a mucocele appendix. Mucoceles can be found in various locations in the body, including the colon and the appendix, and have malignancy potential.

We report a case of a 48-year-old male that presented to the ED with a history of two days of abdominal pain. Upon arriving at the ED, he had a CT scan showing a 9.5 x 4.2 x 6.4 cm fluid collection in the right lower quadrant (RLQ) juxtaposed to the cecum, suggesting appendicitis or an abscess. Initially, a laparoscopic approach was taken, which was then converted to an open laparotomy. The mass was excised and a right hemicolectomy was performed along with an ileocolonic anastomosis due to extensive involvement of the large intestine. Pathology reported a low-grade appendiceal mucinous neoplasm resected with negative margins and 16 negative lymph nodes.

## Introduction

Appendicitis is the inflammation of the appendix, which is located at the tip of the cecum. Appendicitis is mostly encountered between the first and second decade of life and has a male predilection [1]. The mainstay treatment of appendicitis is an appendectomy, which is the surgical excision of the appendix. During an appendectomy, appendiceal masses can be found incidentally. They can be given a collective term such as an appendiceal mucocele, with some being benign while others malignant [2]. Appendiceal mucoceles and neoplasms are rarely encountered during commonly performed acute abdomen surgeries. Due to their rarity, appendiceal mucoceles have historically not been a classification but rather a radiological finding. The Peritoneal Surface Oncology Group International (PSOGI) has alleviated this confusion by creating classifications to aid diagnostic accuracy [3]. The current PSOGI classification breaks down appendiceal mucoceles into two categories: non-neoplastic mucinous and neoplastic mucinous lesions [3]. If appendiceal mucoceles become inflamed, they can perforate. If they are found to be appendiceal neoplasms, they can have peritoneal spread and cause a condition called pseudomyxoma peritonei (PMP) [3]. The subsequent prognosis of the patient depends on the cell type found in the mucin-containing neoplasm. While the PSOGI’s classification has helped clinicians, appendiceal mucoceles are rarely encountered. Their ambiguous clinical and radiological presentations create diagnostic difficulty. In some studies, out of 2660 appendectomies, roughly 0.60% were classified as appendiceal mucoceles [4]. Their occurrence is extremely rare; therefore, they can often be confused with appendiceal abscesses or perforation. Radiological ambiguity can complicate diagnosis and the subsequent course of treatment. Accurate diagnosis is crucial because some appendiceal masses are neoplastic, and their perforation can result in the peritoneal spread of malignancy. We present a low-grade malignant appendiceal mucocele with the hopes of contributing to the clinical presentation of such a condition, thus improving diagnostic and treatment outcomes.

## Case presentation

**Figure 1:**
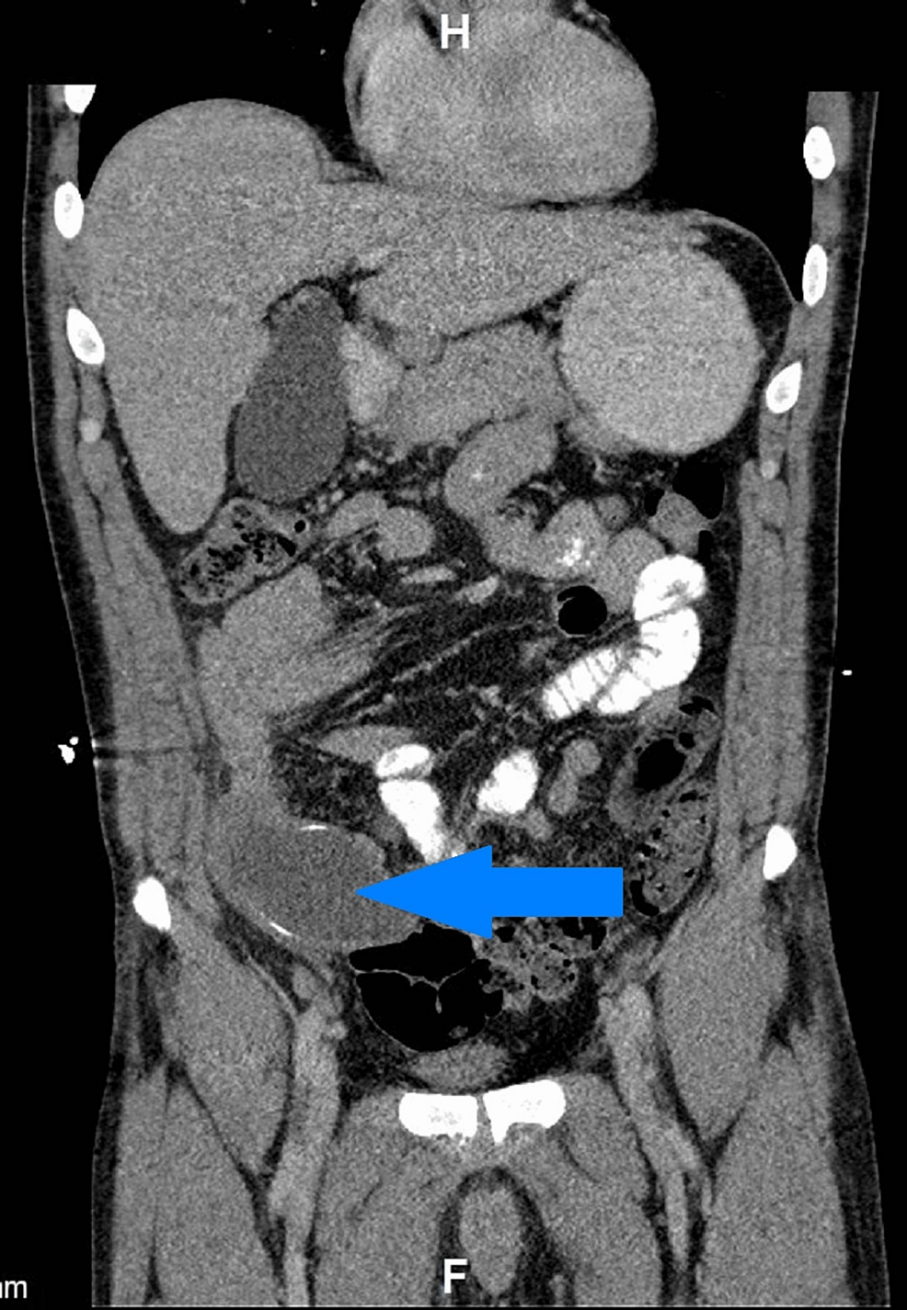
Coronal view of the abdomen with blue arrow pointing to the 9.5 x 4.2 x 6.4 cm appendiceal mucocele.

**Figure 2:**
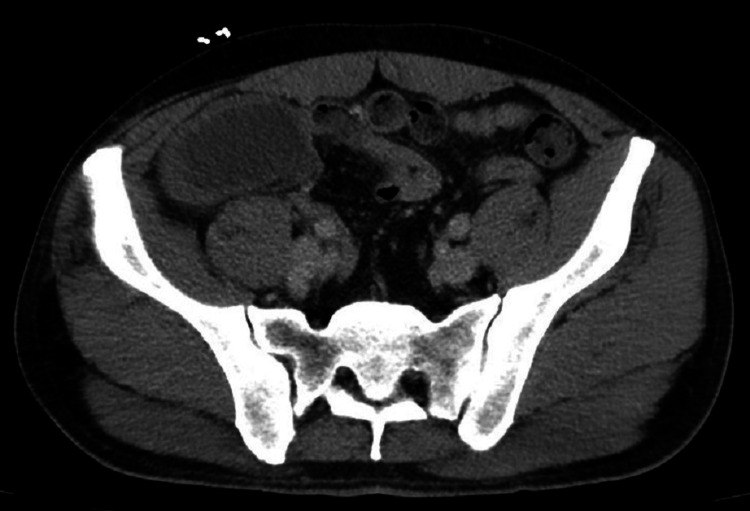
An axial view of the abdomen showing the 9.5 x 4.2 x 6.4 cm appendiceal mucocele.

A 48-year-old male with a two-day history of right lower quadrant pain presented to the emergency department (ED). Upon presenting to the ED, he had an extensive medical workup, including a right lower quadrant (RLQ) CT scan, which showed a 9.5 x 4.2 x 6.4 cm fluid collection in proximity to the appendix shown in axial and coronal view (Figures 1-2). The patient had a history of two surgeries, the only abdominal surgery being a hernia repair. The patient reported a 30-pack per year history of smoking and consumption of a 30-pack of beer weekly. The patient had a history of methamphetamine and marijuana use as recently as a week before presenting to the ED. The patient’s family history was non-contributory. The patient reported weakness, fatigue, and decreased appetite. He was afebrile at admission and remained afebrile for the remainder of his hospital stay. The patient reported abdominal pain and diarrhea, but he denied vomiting. A physical exam in the ED showed diffuse tenderness to palpation with extreme tenderness located at the RLQ. The patient had tenderness at McBurney’s point, located at the RLQ, but he was negative for Murphy’s sign at the right upper quadrant (RUQ). The physical exam was otherwise unremarkable. The patient’s WBC count was tracked during his hospital stay (eight days), with his count returning normal on the day of discharge (Table 1). General surgery was consulted because of this patient’s presentation and laboratory workup.

**Table 1:** WBC count for the duration of the hospital stay. *H = High WBC value **N = Normal WBC value

Day since the ED visit	WBC Count (th/µL)	(Normal range is 4.5-11 th/µL)
Day 1 WBCs	15.4 th/µL (H*)	Day of ED and admission to hospital and IV antibiotics
Day 2 WBCs	17.4 th/µL (H)	
Day 3 WBCs	13.8 th/µL (H)	Day of open appendectomy and right colon hemicolectomy
Day 4 WBCs	15.4 th/µL (H)	
Day 5 WBCs	15.0 th/µL (H)	
Day 6 WBCs	13.9 th/µL (H)	
Day 7 WBCs	12.1 th/µL (H)	
Day 8 WBCs	10.3 th/µL (N**)	Day of discharge from the hospital

Because of this patient's equivocal presentation, interventional radiology was initially consulted to place a percutaneous drain in addition to IV piperacillin and tazobactam. Due to the size of the fluid collection in the RLQ, the radiology department was concerned with possible appendicitis accompanied by an abscess. There was also concern about a possible mucinous neoplasm due to the extensive level of appendiceal distension. Despite IV antibiotics use since ED admission, the patient's WBC count increased from 15.4 th/µL to 17.4 th/µL on day two (Table 1). After the failure to resolve and radiology's uncertainty about the placement of a percutaneous drain, a surgical approach was taken.

The initial surgical approach was minimally invasive, but due to the size of the appendiceal mass, laparoscopic surgery was converted to open right colon resection. Upon opening the abdomen, a large, distended, white appendiceal mass, consistent with a mucocele, was discovered in the RLQ of the abdomen. The mucocele could be likened to the appearance of a white egg enveloping sections of the large bowel. Peri-appendiceal inflammation was also present. The appendiceal mass was resected along with the proximal cecum to the transverse colon. A side-to-side, functional end-to-end, stapled ileocolonic anastomosis was created, between the distal ilium and the proximal transverse colon, via a gastrointestinal anastomosis (GIA) stapler. Fascia and skin were then successfully closed, and dressings were applied to the midline incisions. Blood loss during the surgical procedure was approximately 50 mL. There were no complications or blood transfusions during the surgical procedure. The patient was extubated and taken to recovery in stable condition. Days four through eight of the patient’s stay involved medical and pain management, during which the patient remained hemodynamically stable and asymptomatic.

The pathology report showed low-grade appendiceal mucinous neoplasm surgically resected with negative margins and 16 negative lymph nodes. On day eight, the patient was discharged in a hemodynamically stable state. Eight days post-discharge from the hospital, the patient was seen in the clinic for staples removal. At that time, the patient reported regular bowel movements and flatus. He was instructed to restrain from lifting more than 10 lbs for the next month and advised to follow up with oncology as an outpatient.

## Discussion

Mucocele appendix describes the pathological dilation of the appendix via an obstruction or, more commonly, due to epithelial proliferation. This proliferation can be either benign or malignant [5]. Other conditions that must be part of the differential diagnosis when considering a mucocele appendix are cystadenocarcinoma, carcinoid tumor, and ovarian metastases [6]. While a mucocele appendix is not the rarest mucocele encountered, it only accounts for less than 1% of all surgical appendectomies [7]. Some surgical centers have encountered nine mucocele appendixes in 6000 appendectomies, equating to 0.15% of all surgical cases [7]. A mucocele appendix is most commonly seen due to mucus retention, which can occur via the blockage of the luminal opening of the appendix [8]. However, a simple blockage only results in minimal dilation of the appendix, usually under 2 cm. Mucoceles larger than 2 cm carry a higher chance of malignancy or progression to malignancy; therefore, they must be surgically excised [9].

Due to their radiological ambiguities, complications could arise with the treatment approach regarding mucocele appendiceal masses. There is radiological non-specificity that proves accurate diagnosis is a challenge. While radiological ambiguities exist, on a CT scan, appendiceal mucocele dilation can have lower attenuation than surrounding tissue and can have a homogenous appearance [10]. This was also the case with our patient. This classification challenge due to radiological ambiguity was partially alleviated with the creation of a consensus classification of pseudomyxoma peritonei and associated appendiceal neoplasia by the PSOGI [3]. However, appendiceal mucoceles can have ambiguous presentations creating a difficult clinical picture. This was the situation with our patient as well. If treated incorrectly, it could result in pseudomyxoma peritonei, a malignant process. Similar cases in the past have had a similar clinical course where an accurate diagnosis was only possible after direct visualization of the mass, highlighting the importance of accurate diagnosis to prevent unnecessary treatment and decrease morbidity [7].

Treatment almost always utilizes surgery; however, it can differ depending on the presentation of the mucocele and the chance it is benign or malignant. If the mucocele is smaller in size, then laparoscopic appendectomy can be selected without risking the peritoneal spread of malignancy [11]. An algorithm was created in 2006 by Dhage-Ivatury and Sugarbaker. Depending on radiological appearance and size, it dictates a surgical approach with mucoceles [12]. The algorithm also suggests that a right hemicolectomy be performed when there is a high suspicion of the mucocele being malignant [12]. This was the case with our patient, where a right hemicolectomy was performed with a side-to-side, functional, end-to-end, stapled ileocolonic anastomosis between the distal ilium and the proximal transverse colon via a GIA stapler. The size of the appendix mucocele and the presence of cells in the peritoneum are both essential and poor prognostic factors for a patient's survival. Caution needs to be taken when treating such cases, as was the case with our patient [13].

## Conclusions

An appendiceal mucocele is a rarely encountered surgical case. Its diverse clinical picture makes its diagnosis and treatment challenging even for the most experienced physicians. This was the case with our patient as well. Our patient showcases the ambiguity in the presentation of an appendiceal mucocele. Therefore more case reports in the literature can help standardize the treatment approach and avoid unnecessary complications. While no clear treatment protocol exists, resection with clear margins is favorable for a good prognosis and follow-up with oncology for appropriate treatment. In addition, the luminal size of the mucocele is an important prognostic factor and a crucial factor for surgical approach. Being able to differentiate this condition from a simple abscess that can be drained by interventional radiology is crucial so that the potential spread of malignancy can be avoided. The utilization of multiple standards of treatment and diagnostic criteria can help with accurate diagnosis and treatment, leading to optimal prognoses in patients.
